# Novel approach based on one-tube nested PCR and a lateral flow strip for highly sensitive diagnosis of tuberculous meningitis

**DOI:** 10.1371/journal.pone.0186985

**Published:** 2017-10-30

**Authors:** Yajuan Sun, Jiajun Chen, Jia Li, Yawei Xu, Hui Jin, Na Xu, Rui Yin, Guohua Hu

**Affiliations:** 1 Department of Neurology, the Second Hospital of Jilin University, Changchun, Jilin, China; 2 Department of Neurology, China-Japan Union Hospital of Jilin University, Changchun, Jilin, China; 3 College of Life Science, Jilin Agricultural University, Changchun, Jilin, China; 4 College of Biological Engineering, Jilin College of Agricultural Science and Technology, Jilin, Jilin, China; Institut de Pharmacologie et de Biologie Structurale, FRANCE

## Abstract

Rapid and sensitive detection of *Mycobacterium tuberculosis* (*M*. *Tb*) in cerebrospinal fluid is crucial in the diagnosis of tuberculous meningitis (TBM), but conventional diagnostic technologies have limited sensitivity and specificity or are time-consuming. In this work, a novel, highly sensitive molecular diagnostic method, one-tube nested PCR-lateral flow strip test (OTNPCR-LFST), was developed for detecting *M*. *tuberculosis*. This one-tube nested PCR maintains the sensitivity of conventional two-step nested PCR and reduces both the chance of cross-contamination and the time required for analysis. The PCR product was detected by a lateral flow strip assay, which provided a basis for migration of the test to a point-of-care (POC) microfluidic format. The developed assay had an improved sensitivity compared with traditional PCR, and the limit of detection was up to 1 fg DNA isolated from *M*. *tuberculosis*. The assay was also specific for *M*. *tuberculosis*, and no cross-reactions were found in other non-target bacteria. The application of this technique to clinical samples was successfully evaluated, and OTNPCR-LFST showed 89% overall sensitivity and 100% specificity for TBM patients. This one-tube nested PCR-lateral flow strip assay is useful for detecting *M*. *tuberculosis* in TBM due to its rapidity, high sensitivity and simple manipulation.

## Introduction

Tuberculous meningitis (TBM) is a severe form of central nervous system (CNS) disease that causes substantial morbidity or permanent neurological damage in more than half of those affected in spite of anti-tuberculosis treatment (ATT) [1, 2]. Early and rapid laboratory confirmation of TBM is crucial for successful disease management, and definitive diagnosis of TBM depends upon the detection of *Mycobacterium tuberculosis* (*M*. *Tb*) in cerebrospinal fluid (CSF) [3]. Currently, smear microscopy and culture are most widely used for detecting *M*. *tuberculosis* in CSF. However, smear microscopy is not sensitive, CSF culture methods are time consuming and insensitive in paucibacilliary conditions. Thus, it is unable to provide the rapid and appropriate diagnosis required for proper patient management [2, 4]. Over the past decade, various molecular technologies, particularly polymerase chain reaction (PCR) assays, have been developed as promising new tools for rapid and accurate diagnosis of TBM. The sensitivity of PCR technologies ranges from 55.8% to 87.6% among different measuring methods and laboratories [5–9]. Therefore, the sensitivity of traditional PCR still needs to be improved.

Recently, nested PCR methods have been developed for detection and amplification of *M*. *tuberculosis* DNA with high sensitivity and specificity [10–12]. Nested PCR is a two-step procedure that is performed by first amplifying the target sequence with the outer primers and then using the product as the DNA template for the inner primers for amplification. Although the sensitivity of the detection has increased, there are still some disadvantages, such as an increased risk of carry-over cross-contamination, the complexity of manipulation compared to one-step PCR and the amount of time required. Nested PCR usually takes up to 5–6 hours to complete. Investigators have attempted to develop one-tube or single-tube nested PCR technologies, in which both outer and inner primers are contained in a closed tube, the initial PCR cycles are initialed at high annealing temperatures, and the later cycles are performed at low annealing temperatures, thus reducing carry-over contamination and maintaining high sensitivity[13–20]. However, to date, this technique has been susceptible to inhibition by complex, labor-intensive operation for gel electrophoresis detection or increased equipment cost for real-time fluorescence detection, which greatly diminishes its routine use.

**Fig 1:**
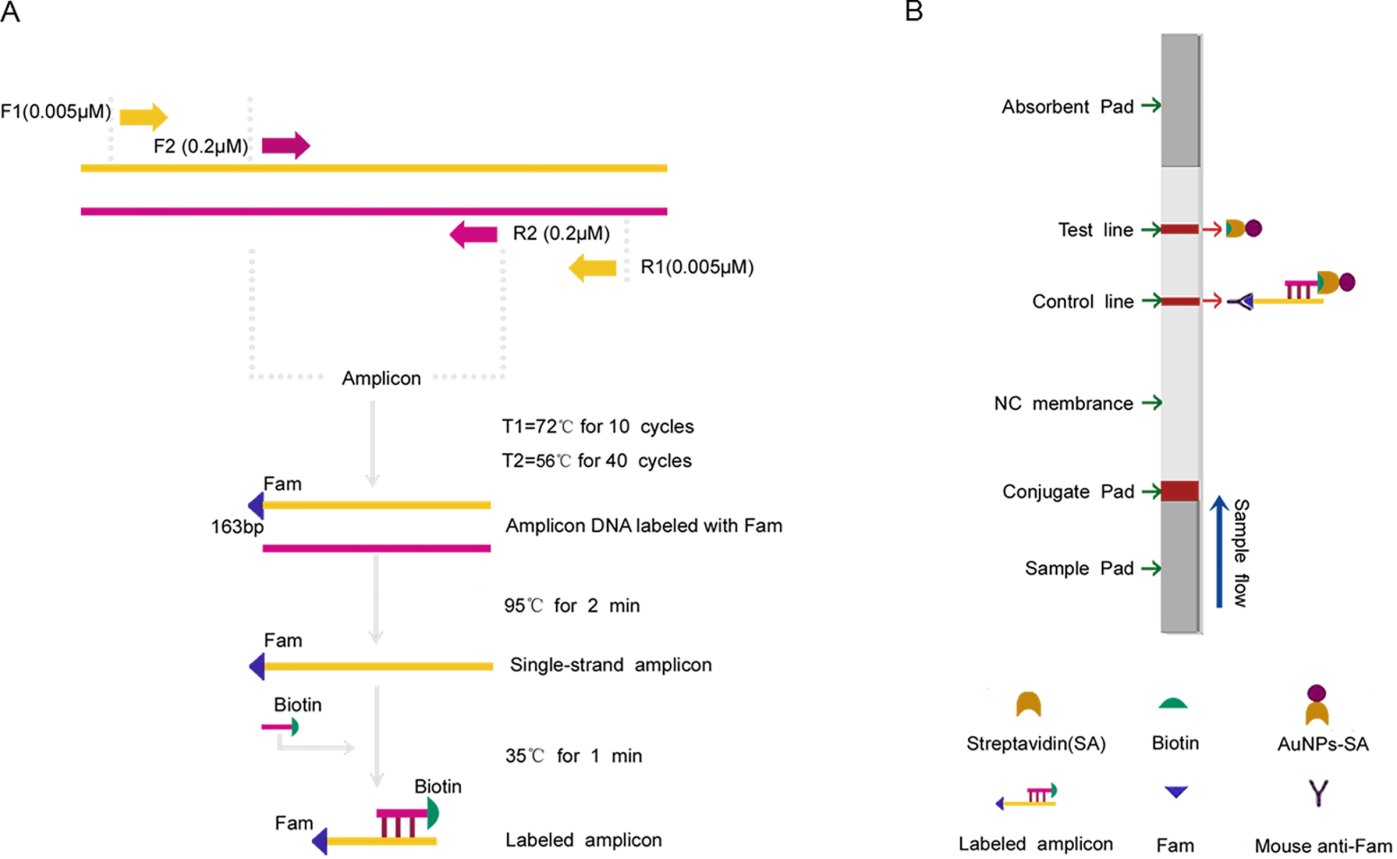
Schematic overview of the OTNPCR-LFBT principle for the detection of *M*. *Tb*. (A) In the present use of the target DNA template, the PCR reaction promoted by the two outer primers (F_1_ and R_1_) will generate amplification product for amplifying by the nest primers (F_2_ and R_2_, F_2_ is labeled with Fam) in a single-tube. The newly generated PCR amplicon is denatured to form single-stranded DNA, the target DNA can be hybridized by the labeled gene-specific probe to generate a sandwich-like product (labeled with Fam and Biotin). (B) After dipping the labeled hybridization products onto the sample pad of the strip, driven by the migration buffer, these products are bound to the colloidal gold particle-streptavidin conjugate on the sample pad of the strip, then migrated in a buffer stream to be captured at the test line (T line) by the mouse anti-Fam antibody, resulting in a visible red line. The excess unbound streptavidin-colloidal particles flow over and are taped at the control line (C line) by Biotin, and the C line is visible. When both the T line and C line are visible, the result is positive. If only the C line is visible, the result is negative.

Most of the earlier studies have used *IS6110* sequence as a target for PCR based diagnosis of TBM [8, 11]. The reason for using *IS6110* is the presence of multiple copies (6–24) in the *M*. *tuberculosis* genome, which gives high sensitivity. In the present study, we developed a highly sensitive molecular test that combines one-tube nested PCR and a lateral flow strip assay for the detection of *M*. *Tuberculosis* in CSF samples, which targeted the *IS6110* sequence and consisted of an uninterrupted reaction in one closed tube. The nested PCR product was hybridized by the labeled gene-specific probe to generate a sandwich-like product (labeled with Fam and Biotin) (Fig 1A). Then, detection was achieved by trapping this labeled product on the surface at the test line of a lateral flow strip, resulting in a visible color signal that can be read by the naked eye within 5 minutes (Fig 1B). This sensitive and easy-to-use system is ideal for further development as a point-of-care (POC) device.

## Materials and methods

### Preparation of positive control

Cultures of *M*. *tuberculosis* H37Ra [there are 18 copies of *IS6110* in the genome of *M*. *tuberculosis* H37Ra (NCBI Reference Sequence: NZ_CP016972.1)] were maintained on modified Roche Medium Base (Qiao Yu Biotechnology Co., Ltd., Shanghai, China). DNA was insolated based on the CTAB-phenol-chloroform method described by Mir AW, et al. (2008) with some modifications [21]. Briefly, the *M*. *tuberculosis* cells were harvested and re-suspended in 200 μL of TE buffer (Tris–EDTA), followed by incubation at 100°C for 10 min, mixing with 30 μL of 10% SDS and 10 μL Proteinase k (20 mg/ml), and finally incubation at 37°C for 1 h. Then, 100 μL of 5 M NaCl and 80 μL of 1.8% CTAB (cetyl trimethyl ammonium bromide) were added and incubated at 65°C for 10 min. After incubation, an equal volume of chloroform: isoamyl alcohol (24:1) was added, mixed thoroughly and centrifuged at 12,000 rpm for 5 min. The supernatant was carefully separated, an equal volume of phenol: chloroform: isoamyl alcohol (25:24:1) was added and then centrifuged at 12,000 rpm for another 5 min. The supernatant was transferred into a new tube and mixed with 500 μL of isopropanol, mixed and spun at 12,000 rpm for 5 min, the supernatant was discarded, and the pellets were washed with 500 μL of 75% ethanol, dried and re-suspended in 25 μL of TE buffer. Other extracted DNA samples from five non-tuberculous mycobacteria strains, *M*. *phlei* (ATCC 607), *M*. *smegmatis* (ATCC 19420), *M*. *gastri* (ATCC 15754), *Escherichia coli O157* (ATCC 35150) and *Staphylococcus aureus* (ATCC 29213) were used in this study.

### Clinical specimen collection and preparation

(i) Clinical specimens. The research was performed retrospectively with blinding in which the clinical diagnosis was not available at the time of the study. Consecutive CSF specimens from a total of 134 patients were collected in China-Japan Union Hospital of Jilin University, from June 2015 to October 2016. The ages of the patients ranged from 15 to 80 years, and the sample included specimens from 74 males and 60 females. Based on the following criteria, the patients were classified into three groups: Group I: confirmed TBM group (n = 34) with a positive CSF smear and/or culture; Group II: suspected TBM group (n = 57) with both a negative CSF smear and culture but with corroborative clinical features of TBM including chronic fever, headache and neck stiffness, response to anti-tuberculosis drugs, CSF criteria were cell count >10 cell/mm3, protein concentration of >40 mg/dL or blood glucose ratio <0.5 [22]. Those patients who did not respond to anti-tuberculosis therapy were excluded from the study. Group III: non-TBM group (n = 43), 29 of whom had viral meningitis, 10 had pyogenic meningitis, and 4 had fungal meningitis based on clinical and laboratory findings, these patients were enrolled as controls. The Institutional Ethics Committee of China-Japan Union Hospital of Jilin University, Changchun, approved this study.

(ii) Processing of CSF samples. First, 4.5–6 mL of CSF was obtained from each patient and divided into two tubes: one sample (0.5–1 mL) for cells, glucose and protein, and another (4–5 mL) for microbiological testing. For conventional bacteriology, CSF aliquots of 3 to 4 mL were concentrated by centrifugation at 3000 × g for 10 minutes, and the sediments were subjected to direct examination by culture and acid-fast bacilli after Ziehl-Neelsen (ZN) staining, respectively. The culture was inoculated onto the modified Roche Medium Base, incubated at 37°C and examined twice per week for 3 months. For DNA specimens used in the PCR, 200 μL of CSF sample was centrifuged at 10,000 × g for 10 min, the sediment was collected, and the *M*. *tuberculosis* DNA was extracted and purified as described above (see preparation of positive control).

### Design of primers and probes and PCR conditions

The primers and probes were designed based on the *IS6110* gene of *M*. *tuberculosis* (GenBank accession number: GU994140.1), BLAST (http://www.ncbi.nlm.nih.gov/blast) was used for checking the specificity of the primers and probe. For one-tube nested PCR, the primers were designed based on the criteria described by Costa J, et al (2012): (i) the external primers should produce a fragment that could be used as the template for the second round of amplification, (ii) the outer primers should be extended to give an annealing temperature at least 10°C higher than the internal primers so that only the outer primers bind the target sequence during the first reactions and (iii) the target sequence should be amplified by both the outer and internal primers at a lower temperature in the second round of reaction[15]. The primers and probe are listed (**Table 1**), and they were synthesized by Sangon Biotech, Dalian, China.

**Table 1:** Primers and probes used in this study.

PCR types	Primer/Probe names	Sequence (5’-3’)	TM (°C)
Conventional PCR	F_2_	Fam-gacacataggtgaggtct	56
	R_2_	aacggctgatgaccaaact	56
	P	aggaccacgatcgctgat-Biotin	59
One-tube nested PCR	F_1_	ctgcgagcgtaggcgtcggtgacaaa	72
	R_1_	ctgaaccgtgagggcatcgaggtggc	72
	F_2_	Fam-gacacataggtgaggtct	56
	R_2_	aacggctgatgaccaaact	56
	P	aggaccacgatcgctgat-Biotin	59

Conventional PCR and one-tube nested PCR were performed using a standard system (Applied Biosystems, Foster City, CA, USA). For the PCR reaction, a typical reaction mix was prepared containing 15 μL of PCR master mix (Qiagen, USA), 1 μL of genomic DNA and 1 μL of each primer and ddH_2_O for a total volume of 25 μL. The PCR amplification conditions for conventional PCR were 95°C for 5 min; 40 cycles at 95°C for 30 s, 72°C for 45 s; and 95°C for 1 min. The conditions for one-tube nested PCR were 95°C for 5 min; 10 cycles of denaturation at 95°C for 30 s and annealing at 72°C for 45 s; 40 cycles of denaturation at 95°C for 30 s, annealing at 56°C for 30 s and extension at 72°C for 30 s; and 95°C for 1 min.

### Construction of the lateral flow strip

(i) Functionalization of streptavidin/gold nanoparticle conjugate. The streptavidin/gold nanoparticle conjugate was prepared based on the method described by Yin, et al. (2016) with some modifications [23]. Briefly, 1 mL of 30-nm colloidal gold nanoparticles (JY-SJ101, Shanghai Jie Yi Biotechnology Co., Ltd.) was adjusted to pH 7.0 using 0.2 M K_2_CO_3_ (A600879, Sangon Biotech, Dalian, China), and 10 μL of 1 mg/ml streptavidin (352623, Rui Jin Biotechnology Co., Ltd, Shanghai) was added and gently vortexed at room temperature for 10 minutes. The mixture was supplemented with 20 μL of 10% (w/v) bovine serum albumin (A600903, Sangon Biotech, Dalian, China) and gently vortexed for 10 minutes, followed by adding 20 μL of 10% PEG20000 (A601790, Sangon Biotech, Dalian, China) into the mixture and gently vortexing for another 10 minutes. The gold mixture was centrifuged at 2,000 × g for 15 minutes at 4°C, the supernatant was removed, and the pellet was re-suspended with 1 mL of a washing buffer (0.05 M Tris and 1% PEG) and gently vortexed for 10 minutes and then centrifuged at 7,000 × g for 15 minutes at 4°C. The AuNPs-SA conjuncts were suspended with 500 μL of a buffer containing 3.03 mg of Tris (VA15382-500g, GenStar Biosolutions Co., Ltd, China), 0.1% PVP (A620435, Sangon Biotech, Dalian, China), 5% sucrose (A100335,Sangon Biotech, Dalian, China) and 0.1% Tween 20 (A600560, Sangon Biotech, Dalian, China). Finally, the AuNPs-SA was impregnated on the absorbent pad of the strip and dried at 37°C overnight.

(ii) Preparation of the lateral flow strip. The lateral flow strip consisted of four components: sample pad, gold nanoparticle conjugate pad, nitrocellulose (NC) membrane and upper absorbent pad. The sample pad was made from glass fiber (GL-b03, JY-BX110) and saturated with a PBS buffer (pH 8.0) containing 0.05% of Tween 20 and 10 mM PBS. Then, the sample was dried at 37°C for 4 h and stored at room temperature. The test line and control line on the nitrocellulose membrane (Millipore 135) were immobilized by dispensing Mouse-Fam antibody (Gei Man Biological Technology Co., Ltd, Guangzhou, China) and BSA-Biotin conjugation (Rui qi Biotech Co., Ltd, Shanghai, China), respectively. The concentrations of mouse-Fam antibody and BSA-Biotin conjugate were 1.0 mg/mL, and the distance between the test and control zones was 0.5 cm. The nitrocellulose membrane was dried at 37°C for 4 h and stored at room temperature. Finally, the sample pad, gold nanoparticle conjugate pad, NC membrane, and absorption pad were assembled on a plastic-backed card. Each part overlapped by 2 mm to ensure that the solution could migrate across the strip during the assay. After assembly, the card was cut into pieces 0.3 cm wide and 7 cm long.

### Optimization of the hybridization conditions

To improve the limit of detection and minimize the nonspecific background of the lateral flow assay, several conditions were systematically studied by comparing the detection performance of the lateral flow strip assay for detecting the target PCR product, including hybridization temperature, probe concentration, hybridization time and composition of the hybridization buffer. To determine the optimal temperature for hybridization, different temperatures (30, 35, 40, 45 and 50°C) were set on the same heat block for hybridization, followed by detection of the hybridization product by the strip. The results were quantitatively measured by the strip read instrument. (see lateral flow strip protocol). The probe concentrations (15, 10, 5, 0.5 and 0.1μM) can only be evaluated when the optimal hybridization temperature was confirmed. Based on the best hybridization conditions confirmed previously, four hybridization times (1, 5, 10 and 20 min) were accordingly studied. To find the best composition of hybridization buffer, different concentration of SSC (2×, 4×, 6× and 8×) and BSA (2%, 4%, 6% and 8%) were also systematically studied.

### Lateral flow strip protocol

The test sample for the lateral flow strip was prepared by adding 10 μL of the PCR product and 1 μL of the probe (10 mM) to 50 μL of hybridization buffer [(4×SSC, 2% BSA), pH7.0] and mixing with a pipette, incubated at 35°C for 1 min. Then, 10 μL of the mixture was added onto the sample pad of the strip, the strip was dipped into 90 μL of the migrating solution (0.01 M PBS, pH 7.4), and the result was read within 5 minute by the naked eye. For quantitative measurements, the strip was put into the strip reader instrument (JY1501GS, Jie Yi Biotech Co., Ltd, Shanghai, China), the ratio of the test line to the control line (T/C) signals measured is proportional to the amount of target molecule.

### Evaluation of the PCR-lateral flow strip assay

For detection limit testing, a serial 10-fold dilution of the genomic DNA of *M*. *tuberculosis* was prepared, ranging from 1 ng to 0.1 fg/reaction, followed by amplification by PCR and detection with the lateral flow strip. The cross-reactivity of the test was analyzed using genomic DNA from *M*. *tuberculosis* and other non-*M*. *tuberculosis* (those listed above), 1 ng of each DNA sample used as the template for the PCR reaction. In total, 134 clinically double-blinded specimens were investigated using the PCR-lateral flow strip assay and compared to smear microscopy and culture.

### Data analysis and statistics

The sensitivity of the PCR-lateral flow strip assay was expressed as percentage (95% confidence interval), positive predictive value and the negative predictive value was calculated using the standard formulae.

## Results

### PCR for *IS6110* gene

**Fig 2:**
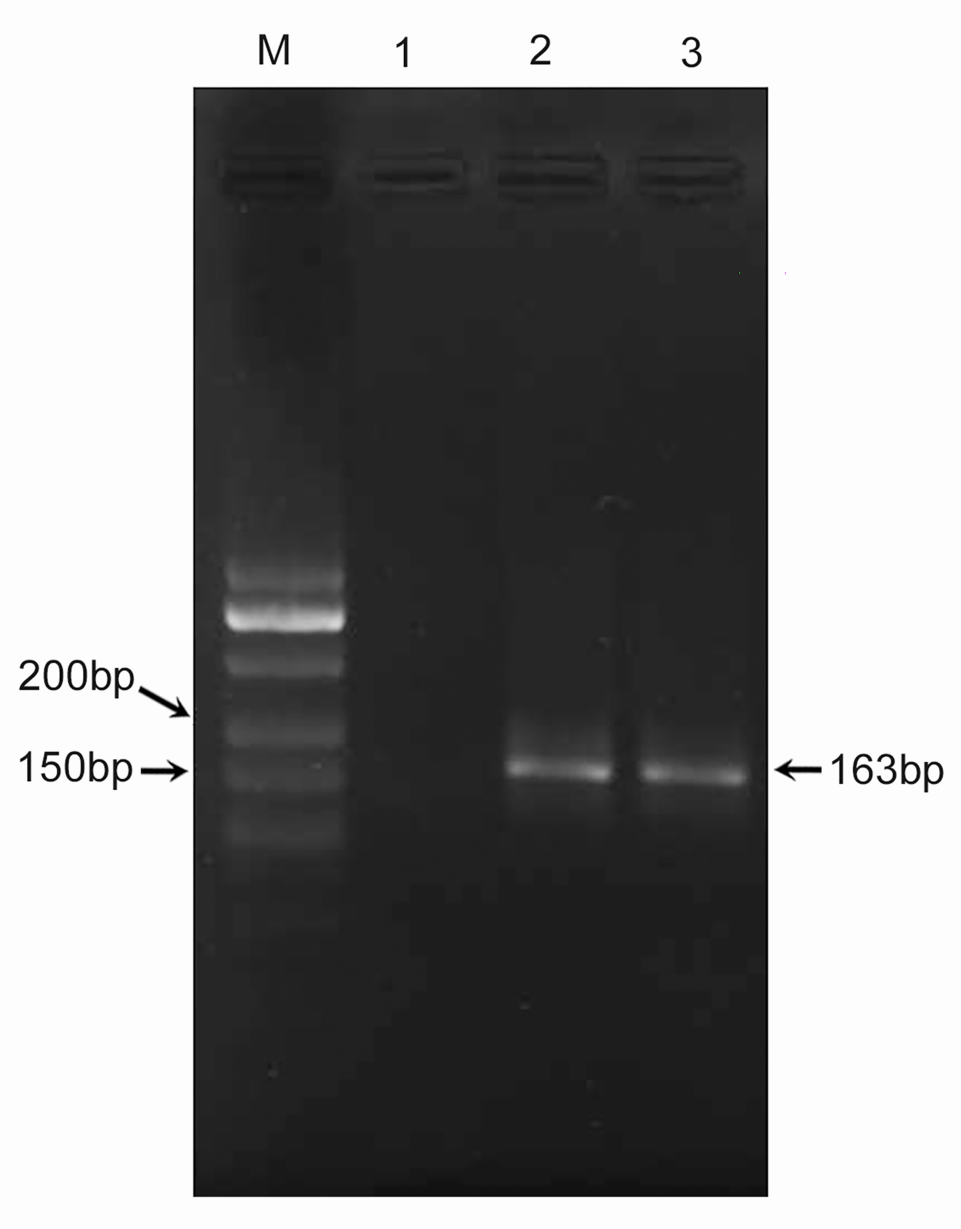
PCR for *IS6110* gene. M: DNA marker; lanes 1 to 3: NTC: no template control contained water, amplification of *IS6110* of *M*. *tuberculosis* using F_2_/R_2_ in conventional PCR, amplification of *IS6110* of *M*. *tuberculosis* using F_1_/R_1_ and F_2_/R_2_ in one-tube nested PCR.

Conventional PCR and one-tube nested PCR were developed using the genomic DNA of *M*. *tuberculosis*. Using the *IS6110* sequence as a target, a 163 bp fragment was amplified by the primer pair F_2_/R_2_ in conventional PCR. Besides that, another 163 bp fragment was also amplified by the primer pairs F_1_/R_1_ and F_2_/R_2_ in one-tube nested PCR (Fig 2). The sizes of these PCR products were expected and were confirmed by sequencing.

Using 1 ng of genomic *M*. *tuberculosis* DNA, several concentrations of primers were investigated. The optimized conditions for conventional PCR were 0.2 μM of each primer (F_2_/R_2_). For one-tube nested PCR, the effect of the ratio of the external primers to internal primers (1: 4, 1: 10, 1: 20 1: 40 1:60 and 1: 80) was also investigated. All six tests generated PCR products with the strongest target bands at 1: 60 (S1 Fig). The optimized conditions for the first round of reactions of one-tube nested PCR were determined with 0.003 μM of each outer primer (F_1_/R_1_), and for the second round of amplifications, the optimized concentration of each inner primer (F_2_/R_2_) was 0.2 μM.

### Optimization of the hybridization conditions

**Fig 3:**
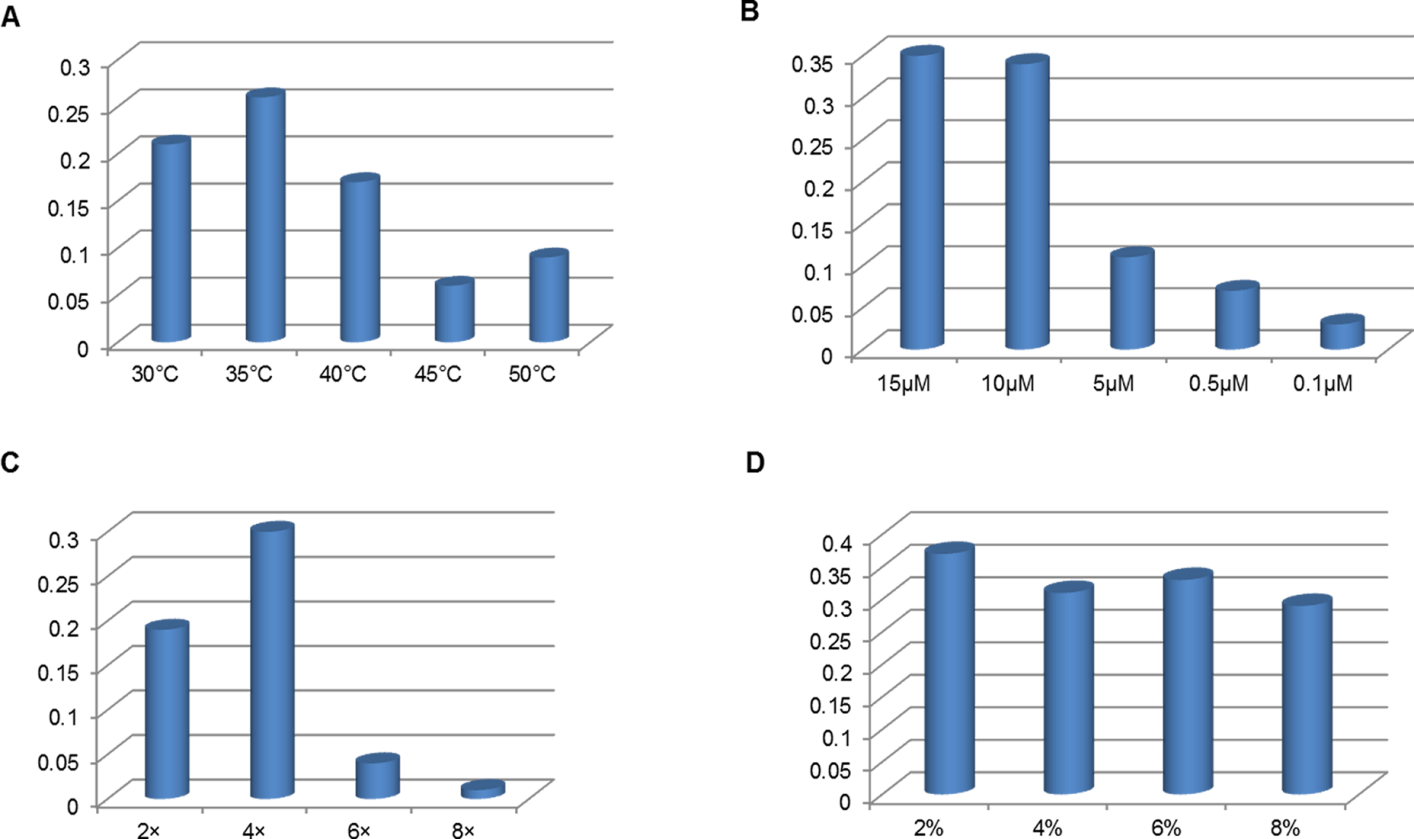
Effect of hybridization conditions on the T /C ratio of the lateral flow strip. (A) Hybridization temperatures, (B) concentration of probe, (C) SSC concentration and (D) BSA concentration.

To obtain the optimal performance, several hybridization conditions were systematically studied. Based on the principal of the quantitative measurements mentioned above, the T/C ratio of strip was the highest when the hybridization temperature was 35°C, thus 35°C was the optimal temperature for hybridization (Fig 3A) The concentration of the probe was set to be 10 μM, and increasing the amount produced no obvious signal change on the T/C ratio (Fig 3B). Hybridization time (1 min, 5 min, 10 min, 20 min) did not obviously influence the performance of the probe hybridization. Thus, 1 min was selected for hybridization (data not shown). To promote the hybridization efficiency of single strain DNA and probe, the composition of the hybridization buffer was also investigated. The best result was obtained with the 4×SSC matrix and 2% of BSA (Fig 3C and 3D). Under optimal hybridization conditions, one test can be finished 5 minutes after PCR.

### Assay development

**Fig 4:**
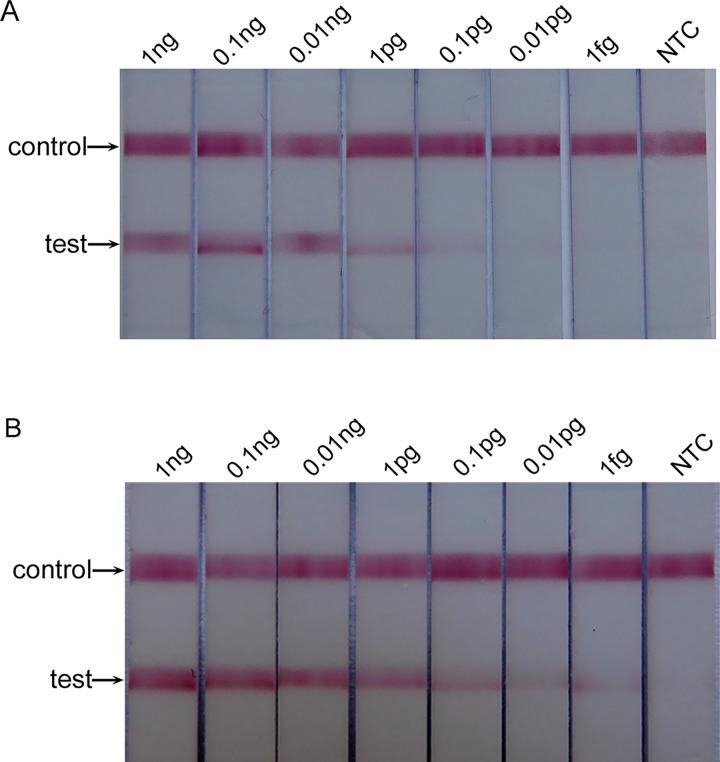
**The detection limit of CPCR-LFBT (A) and OTNPCR-LFBT (B)**. NTC: no template control contained water.

The detection limit of the conventional PCR-lateral flow strip test (CPCR-LFST) and OTNPCR-LFST were evaluated using a serial 10-fold dilution of H37Ra *M*. *tuberculosis* genomic DNA. CPCR-LFST was able to detect 0.1 pg of *M*. *tuberculosis* DNA, whereas OTNPCR-LFST could detect as few as 1 fg of *M*. *tuberculosis* DNA (Fig 4). Thus, in the present study, the OTNPCR-LFST was 100 times more sensitive than CPCR-LFST.

**Fig 5:**
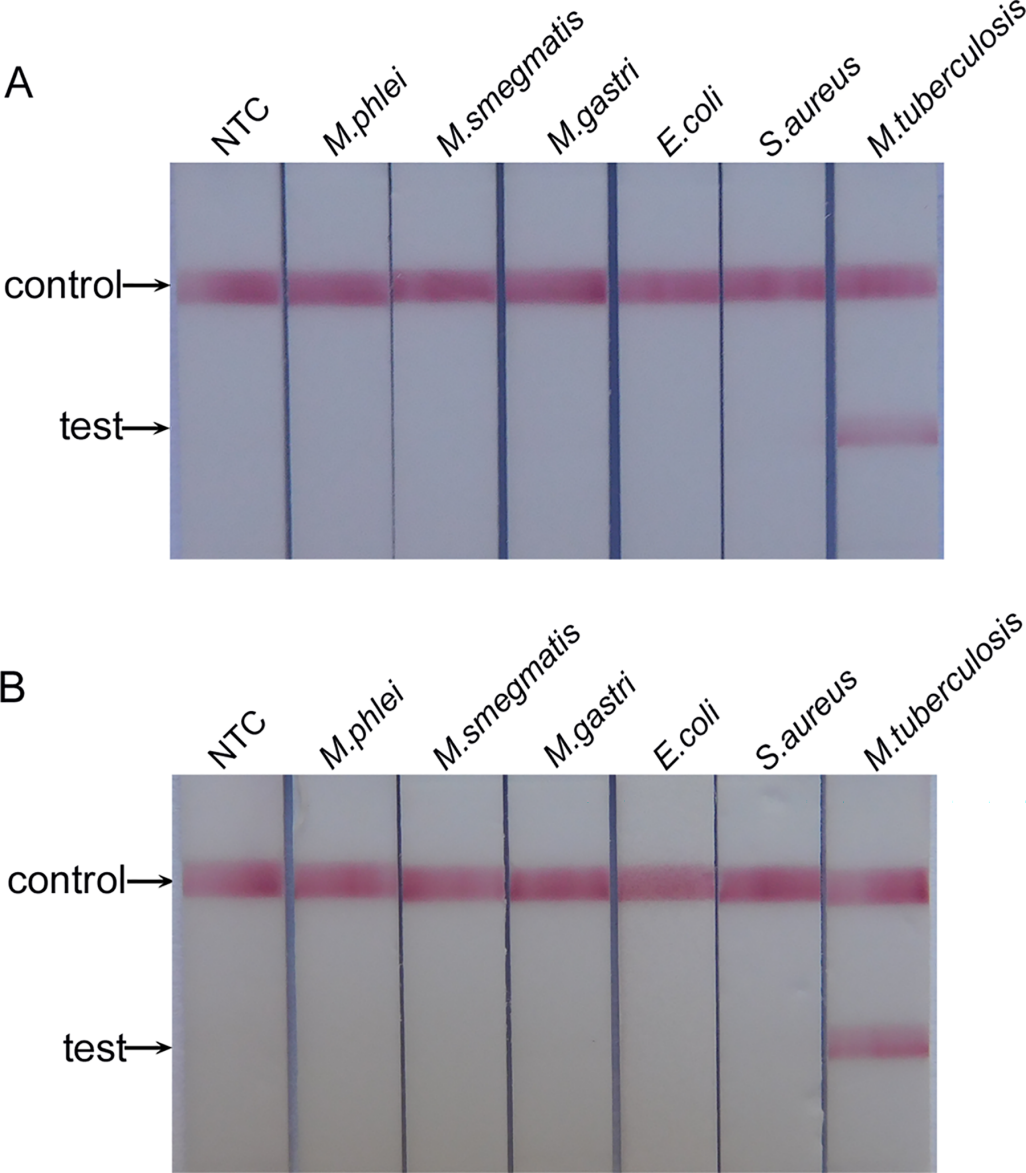
**The cross-reactivity analysis of CPCR-LFBT (A) and OTNPCR-LFBT (B)**. NTC: DNA extracted from the CSF of healthy person.

To evaluate the cross-reactivity of the test, several purified DNA samples of *M*. *tuberculosis* and other non-*M*. *tuberculosis* strains were tested. Using the DNA extracted from the CSF of a healthy person as the negative control, both CPCR-LFST and OTNPCR-LFST showed high specificity, and no cross-reaction was found against other non-target bacteria (*M*. *phlei*, *M*. *smegmatis*, *M*. *gastri*, *Escherichia coli O157*, *Staphylococcus aureus*) (Fig 5).

### Clinical validation

A total of 134 clinical, double-blinded CSF samples were used to evaluate the performance of the PCR-lateral flow strip assay. The results of different diagnostic tests for TBM for these patients are shown in **Table 2.** The smear examination was positive in ten cases, which were also positive by culture, CPCR-LFST and OTNPCR-LFST. *M*. *tuberculosis* culture was positive in 34 patients, CPCR-LFST in 71 patients, and OTNPCR-LFST in 81 patients. Using culture as the gold standard, 88.2% (95% CI 81.8%-94.7%) and 97.1% (95% CI 93.8%-100.4%) were detected as positive by CPCR-LFST and OTNPCR-LFST, respectively. However, in the clinically diagnosed TBM cases that were not confirmed by smear/culture, the sensitivity of CPCR-LFST and OTNPCR-LFST were 71.9% (95% CI 60.1%-83.7%) and 84.2% (95% CI 74.8%-93.6%), respectively. The overall sensitivity (including the confirmed TBM and suspected TBM cases) of CPCR-LFST and OTNPCR-LFST were thus found to be 78.0% (95%CI 69.4%-86.6%) and 89.0% (95% CI 82.4%-95.4%), the positive predictive values are both 100%. In the non-TBM group, all the tests were negative, the specificity of the CPCR-LFST and OTNPCR-LFST were both 100%, the negative predictive values are 68.3% and 81.1%, respectively

**Table 2:** Result of smear, culture, CPCR-LFST and OTNPCR-LFST.

	Smear N (N%)	Culture N (N%)	CPCR-LFST N (N%)	OTNPCR-LFST N (N%)
Confirmed TBM (n = 34)	10 (29.4)	34 (100.0)	30 (88.2)	33 (97.1)
Suspected TBM (n = 57)	0 (0.0)	0 (0.0)	41 (71.9)	48 (84.2)
Total (n = 91)	10 (11.0)	34 (37.4)	71 (78.0)	81 (89.0)
Control group (n = 43)	0 (0.0)	0 (0.0)	0 (0.0)	0 (0.0)

## Discussion

The present study provided a simple molecular method that combines a one-tube nested PCR assay with a lateral flow strip for diagnosis of TBM in patients. Using the *IS6110* gene as the target, the concentration of each primer, the reaction parameters of the PCR and hybridization conditions were systematically optimized. Under the optimal conditions, the detection limit of the OTNPCR-LFST was up to 1 fg of *M*. *tuberculosis* genomic DNA, which is less than the amount of DNA in a single bacterial cell (approximately 5 fg) [24]. In the present study, this OTNPCR-LFST was 100 times more sensitive than CPCR-LFST, which is consistent with previous reports showing that nested PCR greatly increases sensitivity compared to traditional PCR [13–15]

The performance of the OTNPCR-LFST was further evaluated using 134 clinical specimens. The assay had a positivity rate of 89.0% among TBM patients, which was higher than CPCR-LFST (78.0%), CSF microscopy (11%) and *M*. *tuberculosis* culture (37.4%). CSF characteristics and clinical data of culture-negative/OTNPCR-LFST-positive cases were in line with a diagnosis of TB meningitis, and specificity of the OTNPCR-LFST was 100% in the non-TBM group. The reason for OTNPCR-LFST negativity in 10 out of 91 clinically diagnosed TBM cases could be the presence of the low number of bacteria or insufficient lyses of bacteria, or possibly effected by the presence of some PCR inhibitors in the samples.

Several pervious studies have evaluated conventional two-step nested PCR targeting *IS6110* for diagnosis of TBM patients, with a high sensitivities of 75% and 98%, respectively [11, 25], while the sensitivity of OTNPCR-LFST was 89% for TBM patients. Based upon the findings of the previous reports, we can suggest that the OTNPCR-LFST retained the sensitivity of the conventional two-step nested PCR while simplifying the test procedures and reducing the risk of cross-contamination and labor required. In the detection process, the result can be readout on a strip by the naked eye with no requirement of trained personnel and special equipment, thereby increasing access in resource-limited settings. OTNPCR-LFST shows clear potential for implementation as a reference diagnostic tool in midlevel-equipped laboratory facilities (with common PCR instruments and power) and will be of particular value for early, rapid and accurate diagnosis of TBM in clinical patients.

There are still several limitations that must be addressed in follow-up works. In the present study, the *M*. *tb* genomic DNA extraction still involved several manually performed steps that are not user friendly. There is still the need to open the tube after PCR amplification for detection, which increases the risk of amplicon carry-over. Thus, it is not ready to be used as a POC technology in resource and technology-limited settings. For POC use, it is imperative to develop a sample-in to answer-out system that combines sample preparation, PCR reaction and lateral flow detection in an integrated and automatic system. In a parallel effort, we are developing a simple DNA extraction subunit based on similar design principles, which performs DNA isolation in a lysis microreactor [26] and lateral flow detection in a disposable, closed system [27]. Once an automated sample-in to answer-out diagnostic platform is developed, this method could eventually be used at POC settings.

In conclusion, in the present study, a one-tube nested PCR combined with a lateral flow strip test was developed for fast and accurate detection of *M*. *tuberculosis* DNA. This one-tube nested PCR protocol maintains the sensitivity of conventional two-step nested PCR while reducing both the chance of cross-contamination and the time used for analysis. In addition, this newly developed assay was successfully evaluated with clinical specimens, and with further development, this accurate and easy-to-read format could serve as part of POC diagnosis of TBM in resource-limited settings.

## Supporting information

S1 Fig Optimization of the outer/inner primers concentration ratios. Lanes 1 to 8: DNA marker, no template control, 1: 4, 1: 10, 1: 20 1: 40 1:60 1: 80.
(TIF)
