# Supplementary figures and images for: Novel approach based on one-tube nested PCR and a lateral flow strip for highly sensitive diagnosis of tuberculous meningitis
Source: PLoS One. 2017 Oct 30;12(10):e0186985. doi: 10.1371/journal.pone.0186985 (PMC5662171; doi:10.1371/journal.pone.0186985)

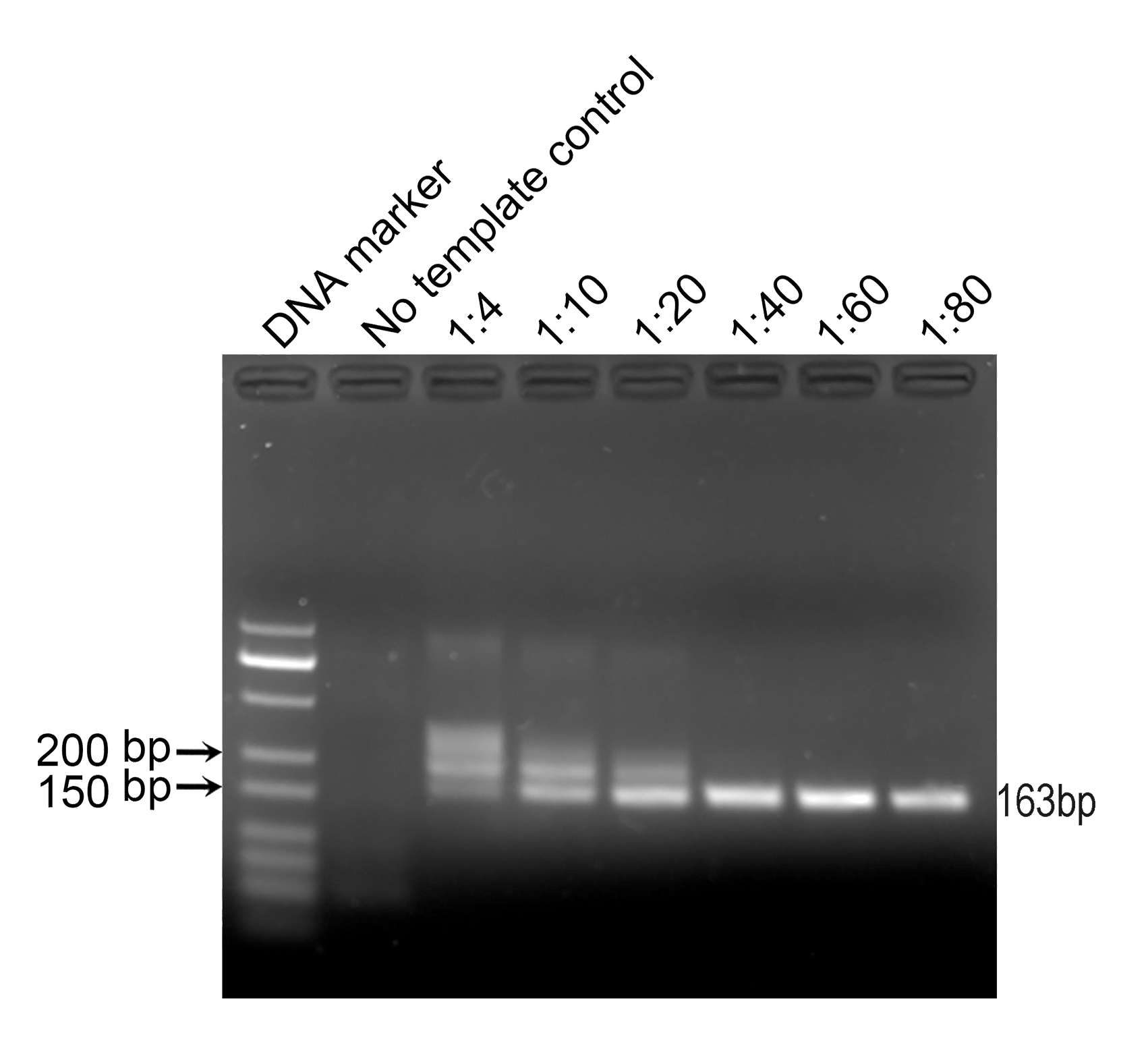

Supplement: S1 Fig — Lanes 1 to 8: DNA marker, no template control, 1: 4, 1: 10, 1: 20 1: 40 1:60 1: 80. (TIF) [file pone.0186985.s001.tif]
